# Multidimensional Decomposition and Ensemble Modeling of Histatin 1 and Its Siblings: Detailing Structure and Biological Function Using an Integrative Approach

**DOI:** 10.1021/acs.jcim.5c00854

**Published:** 2025-07-02

**Authors:** Oskar Svensson, Yuri Gerelli, Marie Skepö

**Affiliations:** † Division of Computational Chemistry, Department of Chemistry, Science for Life Laboratory, 90825 Lund University, P.O. Box 124, SE-221 00 Lund, Sweden; ‡ NanoLund, 9327 Lund University, Box 118, 22100 Lund, Sweden; § CNR—Institute for Complex Systems, Piazzale Aldo Moro 2, 00185 Roma, Italy; ∥ Department of Physics, Sapienza University of Rome, Piazzale Aldo Moro 2, 00185 Roma, Italy

## Abstract

Histatins are a family of multifunctional, cationic histidine-rich saliva peptides. The most prominently represented are Histatin 1, Histatin 3, and Histatin 5. Despite considerable similarities in primary structure, the three members are known to display varied antimicrobial properties and healing abilities. This study aims to provide a detailed structural comparison of Histatin 1, Histatin 3, and Histatin 5, as well as a thorough investigation into the variation caused to the conformational ensemble of Histatin 1 upon phosphorylation. The study applies molecular dynamics simulation, small-angle X-ray scattering, circular dichroism, bioinformatics tools, and neutron reflectometry. A multidimensional decomposition technique and its connection to clustering methods are also presented. It was observed that the phosphorylation of Histatin 1 profoundly shifts the conformational ensemble and may act as a molecular switch that facilitates tooth enamel binding. Observations are provided on the killing mechanisms of Histatins concerning self-association and membrane rupturing.

## Graphical abstract

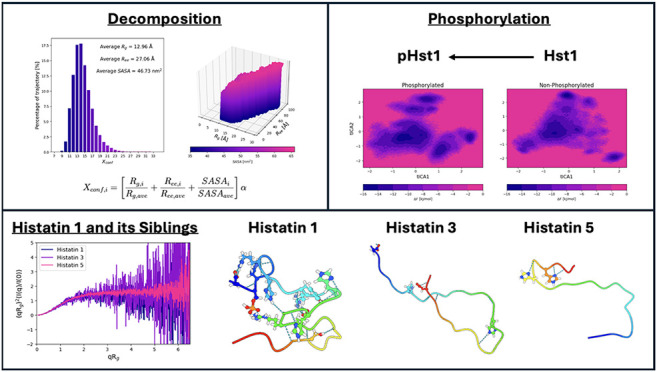

## Introduction

The human oral cavity represents a critical interface between the body and the external environment, constantly exposed to microbial pathogens. Saliva, a biologically complex fluid, plays a pivotal role in innate immunity, forming the first line of defense against bacterial, fungal, and viral infections.^1,2^ Among the numerous bioactive components of saliva, Histatin peptides (Hsts) stand out as multifunctional, cationic proteins with potent antimicrobial properties. These histidine-rich peptides, particularly Histatin 1 (Hst1), Histatin 3 (Hst3), and Histatin 5 (Hst5), have garnered significant attention due to their distinct antimicrobial functionalities.^2–4^ Hsts are also involved in the formation of acquired tooth pellicle. Hst1 is subject to phosphorylation on its Ser2 position; this phosphorylated state (pHst1) aids in the binding to tooth enamel.^2,5^ Hsts are well-known for their antifungal capabilities, with potency against .^2,6,7^ Hst5 is generally described as the most potent, followed by Hst3 and then Hst1.^2^ Hsts are also antiviral; however, less is known about this property.^2–4^ Interestingly, Hst1 and Hst3 have been stated as being more potent than Hst5. These findings are surprising, given Hst1’s typical role in cell activation and its high sequence similarity to the more antimicrobial Hst5.^3,5,7,8^ Such an oxymoronic dynamic provides an excellent system for further detailing the structure of medicinally interesting biomolecules. In our previous publication, an analysis method for decomposition was introduced.^9^ This approach involves sorting simulated conformations based on a system parameter, such as the radius of gyration (*R*_g_). Decomposition provides a simulation-specific option to organize data. The method is highly interpretable and directly connected to the system’s underlying physics. Machine learning methods, such as clustering, can capture immense complexity with the added caveat of hyperparameter tuning and a need for careful selection according to data. We believe that decomposition could prove a useful complement. In the present study, we aim to elucidate the structural dynamics of Hsts with an additional focus on the effect of phosphorylation on Hst1, employing experimental and computational techniques coupled with advanced data decomposition strategies. The intention is to demonstrate further the decomposition technique’s application and its intermingling with machine learning-based methods while introducing multivariable decomposition. An integrated approach will be applied, including the use of small-angle X-ray scattering (SAXS) and circular dichroism (CD) as experimental validation for molecular dynamics (MD) simulations, along with bioinformatics tools and neutron reflectometry (NR).

## Methodology

### Small-Angle X-ray Scattering Measurements

Synthesized peptide powder, purchased from TAG Copenhagen A/S, Denmark, with a purity of 95%, was dissolved in 150 mM NaCl, 20 mM Tris, pH 7.5 buffer, and cleaned through centrifugation dialysis. Centrifugation was applied via Vivaspin2 2000 MWCO membrane centrifugation tubes. SAXS measurements were conducted at the BM29 beamline at the European Synchrotron Radiation Facility (ESRF) in Grenoble, France. All measurements were performed at an energy of 12.5 keV and a temperature of 20 °C with a *q*-range of 0.00776–0.495 Å^–1^. Ten frames were collected for each sample; frames affected by radiation damage were discarded before averaging. The buffer was measured before and after sample measurements. The frames were then handled similarly to the sample frames. Buffer data sets were subtracted from corresponding sample data sets before analysis. Rebinned data for χ2 calculations was created via joining every ten data points, this was done using DATREGRID, part of the ATSAS package.^10^ Analysis was carried out using PRIMUS, also a part of the ATSAS package. *R*_g_ was determined from spectra using the Guinier approximation with a *qR*_g_ limit of 1.1.^11^ Previously published spectra were used for Hst5.^12^ The concentration of the peptide samples was measured using a Nanodrop 2000 instrument.

### Circular Dichroism Measurements

Synthesized peptide powder, purchased from TAG Copenhagen A/S, Denmark, with a purity of 95%, was dissolved in 150 mM NaF, 20 mM sodium phosphate, pH 7.0 buffer, and cleaned through centrifugation dialysis. Centrifugation was applied via Vivaspin2 2000 MWCO membrane centrifugation tubes. White precipitation was formed upon the dissolution of Hst1, which significantly lowered the peptide concentration within the samples. The measurements were conducted at the ASTRID2 beamline at the Centre for Storage Ring Facilities in Aarhus (ISA), Denmark. The protein concentrations were measured using a Nanodrop 2000 instrument.

### Neutron Reflectometry

NR measurements were performed on FIGARO,^13^ a time-of-flight reflectometer at the Institut Laue-Langevin (ILL) in Grenoble, France. The measurements were performed at the solid–liquid interface, using 8 × 5 × 1.5 cm^3^ silicon single crystals cut along the 111 plane and polished to 5 Å root-mean-square (RMS) roughness (Siltronix ST, Archamps, France). They were cleaned by soaking in an ultrasonic bath using, in series, chloroform, acetone, and ethanol. The reflectometer was configured to operate with incident wavelengths ranging from 2 to 20 Å and at two angles of incidence, 0.8 and 3.0°, resulting in a *q*-range of 0.0045–0.3 Å^–1^. To apply the contrast variation method, a series of buffers with varying volume ratios of H_2_O and D_2_O were used, these being 100% H_2_O (HBuf), 100% D_2_O (DBuf), 38/62 DBuf/HBuf ratio (SiMB) having a scattering length density (SLD) matching that of crystalline silicon (2.07 × 10^–6^ Å^–2^), and 66/34 ratio DBuf/HBuf (4MBuf), with an SLD value of 4 × 10^–6^ Å^–2^. Detector images were converted to reflectivity curves, *R*(*q*), using the COSMOS routine provided by the ILL.^14^ The clean silicon substrates were characterized for all of the measurements in both DBuf and HBuf. Solid-supported lipid bilayers (SLBs) were prepared by exploiting the vesicle fusion method following the protocol described in ref 36. SLBs were then characterized before and after the injection of peptides, the latter step being performed at a concentration of 1 mg mL^–1^.

### Atomistic Simulations

Atomistic MD simulations were performed with the GROMACS package version 2021.^15,16^ All simulations applied the AMBER99SB-ILDN force field with the TIP4P-D water model.^17,18^ The chosen combination has been extensively tested and used on similar systems in previous research.^19^ Applied phosphoserine parameters were created by Homeyer et al.^20^ The single chain of pHst1, Hst1, Hst3, and Hst5 was simulated separately by being inserted into a dodecahedron box with a minimum distance of 1 nm from the box edges. Periodic boundary conditions were applied in all directions. Starting configurations for all simulations consisted of a linear chain created in Avogadro.^21^ Both termini were modeled in their charged states, and side chains were assigned according to neutral pH conditions. Histidine residues were treated as uncharged. This resulted in net charges of 0 for pHst1, +1 for Hst1, and +5 for both Hst3 and Hst5. NaCl generally replaced solvent molecules to attain a salt concentration of 150 mM and keep electrostatic neutrality. The primary structures of Hst1, Hst3, and Hst5 are given below in Figure 1. Phosphorylation of Hst1 occurs at the Ser2 position.^5^

**Figure 1:**
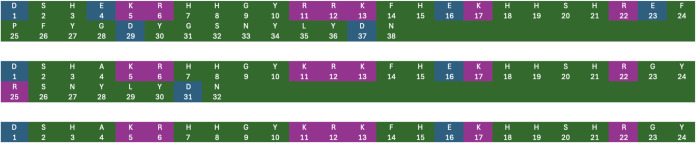
Sequences for Histatin 1 (top), Histatin 3 (middle), and Histatin 5 (bottom). Positively charged residues are colored purple, and negatively charged residues are colored blue.

GROMACS’ leapfrog integration algorithm was used for the equations of motion with a 2.0 fs time step. The Verlet cutoff scheme was applied for nonbonded and short-ranged interactions with a cutoff of 12 Å. Dispersion corrections were applied to energy and pressure. The particle-mesh Ewald (PME) method^22^ was used for long-ranged electrostatics with cubic interpolation and grid spacing of 1.6 Å. Bonds containing hydrogen were constrained using the LINCS algorithm.^23^ The temperature was set to 298 K, applying the Noose–Hoover thermostat^24^ with a temperature fluctuation time of 1.0 ps. A separate coupling group was used for the peptide. The pressure was set isotropically by applying the Parrinello–Rahman barostat.^25^ Pressure coupling used a time constant of 5.0 ps and compressibility of 4.5 × 10^–5^ bar^–1^. Energy minimization used the steepest-descent algorithm. Equilibration was carried out in three steps: (i) 0.5 ns in the *NVT* ensemble, (ii) 0.5 ns in the *NPT* ensemble, and (iii) 1.0 ns in the *NPT* ensemble, where *N*, *P*, *V*, and *T* correspond to a constant number of particles, pressure, volume, and temperature, respectively. Position restraints were used for the peptide during all equilibration steps. Five trials were simulated with a production run of 2 μs each; all simulated systems totaled 10 μs. The Hst1 simulations were the exception; the trials were roughly 1.5 μs each, totaling 7.5 μs. Convergence was determined by checking the equilibration of *R*_g_; see Figures S2–S5 in the Supporting Information. The Hst5 simulations are previously published.^9^

### Analysis Description

GROMACS software^15,16^ provided tools for several analysis methods. gmx pairdist was used to calculate the minimum pair distance, contact maps, and gmx hbond, the hydrogen bond contact maps. The end-to-end distance (*R*_ee_) was calculated using gmx polystat and gmx sasa for solvent accessible surface area (SASA). gmx rmsf was used to calculate root-mean-square fluctuations (RMSF). gmx polystat and gmx gyrate were used to calculate *R*_g_. The Dictionary of Secondary Structure of Proteins (DSSP) algorithm^26^ was run within GROMACS using gmx dssp. Polymer shape (*P*_S_) was calculated using eq 1. Decompositions^9^ were performed using homemade Python scripts. All analyses on nondecomposed trajectories applied a third of all simulated frames from said trajectory. AIUPred^27^ was used to gauge disorder probability. Trans-membrane tendency and hydrophobicity were determined using the ProtScale Web server.^28^ A CIDER analysis^29^ was carried out using the wild-type sequences of Hst1, Melittin, LL-37, Magainin 2, and Cecropin A.

1 $$P_{S}=\frac{R_{\mathrm{ee}}^{2}}{R_{g}^{2}}$$

Example structures were extracted by performing principal component analysis (PCA) and *K*-means clustering via Scikit-learn.^30^ φ–ψ angles were used as input data for dimensionality reduction; these angles were calculated using MDtraj.^31^ The frame numbers within each cluster were then extracted using a homemade Python script and turned into frame index files used to run the gmx cluster command. This then produced average snapshots visualized using ChimeraX.^32^ CRYSOL^33^ was used to determine theoretical SAXS spectra from simulations, with a contrast of hydration shell value set to 0; all other parameters used default settings. χ^2^ was calculated using eq 2, where *E_i_* designates an experimental data point and *S_i_* each a simulated data point, and *N* is the total number of points in one data set. Applying eq 2 assumes an identical number of data points in both simulated and experimental data sets. Rebinned SAXS data were used for χ^2^ calculations, the spectra were normalized by I(0) in preparation for χ2 calculations.

2 $$\chi^{2}=\sum_{i=1}^{N}\frac{{\left(E_{i}-S_{i}\right)}^{2}}{S_{i}}$$

Free energy landscapes were determined via eq 3,^34^ where Δ*F* is the free energy, *k*_B_ is the Boltzmann constant, *T* is the temperature, *n*_*i*_ is a specific bin in a 2D histogram, and *n*_max_ is the most populated bin in a 2D histogram. The 2D histogram is created from the frames of a simulation, represented by two parameters. In this case, φ–ψ angles in each frame underwent time-lagged independent component analysis (tICA) dimensionality reduction with a lag time of 50, whose principal components were then used for the 2D histogram. Matplotlib^35^ was used to create a 2D histogram. The landscapes obtained from eq 3 are normalized by subtracting the energy value of the highest energy state, setting the said state to zero.

3 $$\Delta F=-k_{B}T\ln\left(\frac{n_{i}}{n_{\max}}\right)$$

## Results and Discussion

### Comparison of Histatin Peptides

#### Structural Properties Obtained through Small-Angle X-ray Scattering

SAXS measurements were carried out on pHst1, Hst3, and Hst5. The resulting spectra are displayed in Figure 2. There exists a clear indication of intrinsically disordered behavior for all three peptides. Disordered behavior is most prominently seen in the high intensity observed at high *q* values within the Kratky plots. The Kratky plots are further scrutinized in Figure 2g, where there appears to be no significant difference in the degree of disorder. However, pHst1 could adopt more globular conformations, since the Kratky plot shifts toward lower intensity at high *q* values. Clear evidence of disorder exists in Hst5.^9,12^ However, a significant reduction of published evidence occurs in the case of Hst3 and pHst1.^36^ In all three cases, simulated spectra match decently well with the experiment, with intensity plot χ^2^ values of 2.22, 1.31, and 1.69 for pHst1, Hst3, and Hst5, respectively. Experimental *R*_g_ was determined to be 15.8, 13.8, and 13.4 Å for pHst1, Hst3, and Hst5, respectively. These values are compared to the simulated average *R*_g_ of 12.96, 14.05, and 13.16 Å. Experimental and simulated *R*_g_ generally agree well. However, the simulated *R*_g_ for pHst1 implies that the model generates more compact conformations on average. Such shortcomings in the model have been documented before.^37^ This is also seen in the simulated Kratky plot in Figure 2d.

**Figure 2:**
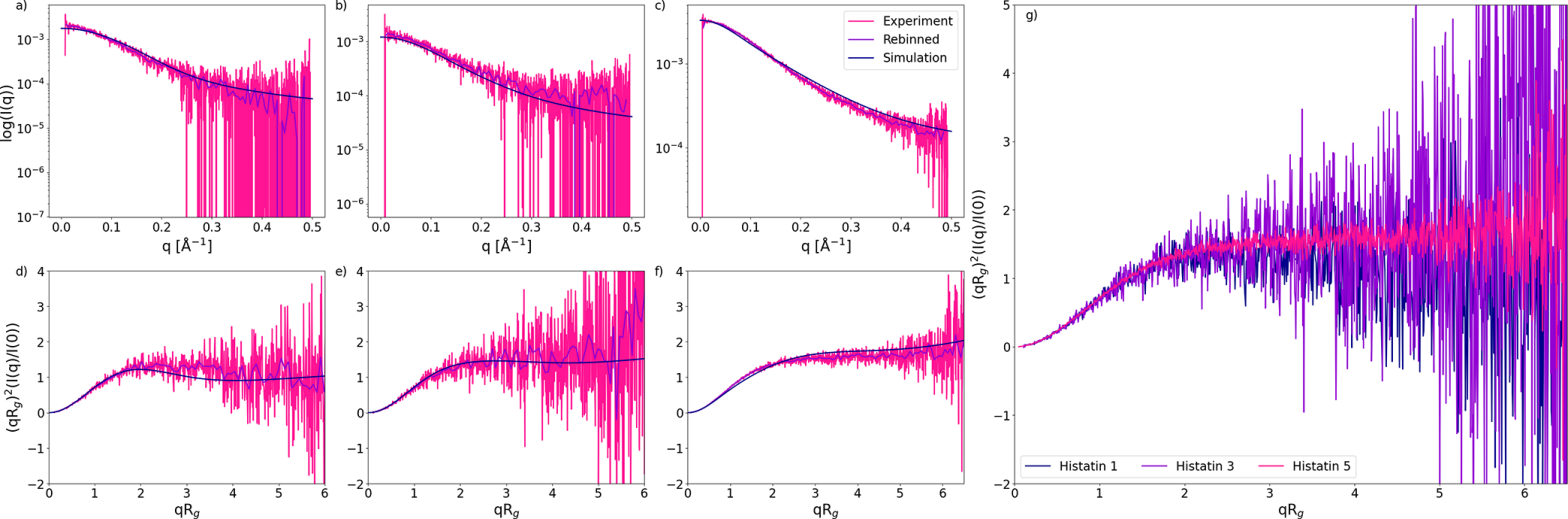
Small-angle X-ray scattering spectra for Histatin 5 (c, f), Histatin 3 (b, e), and phosphorylated Histatin 1 (a, d); both experimental and simulated spectra are shown. The intensity, *I*(*q*), as a function of the scattering vector, *q*, on a semilogarithmic scale (a–c) and normalized Kratky plots, (*qR*_g_)^2^(*I*(*q*)/*I*(0)), as a function of *qR*_g_, (d–f) are displayed. Kratky plot comparison between the three peptides (g). Note that spectra have been normalized in *q* = 0.2.

#### Circular Dichroism and Secondary Structure

CD was measured at multiple concentrations of pHst1, Hst3, and Hst5. Figure 3a–c indicates disordered behavior due to the negative bands around 200 nm. There are signs of poly-proline II (PPII) helix elements, with positive bands centered around 225 nm; these bands are further scrutinized in Figure 3d. The amount of signal varies with chain length, where Hst5 exhibits the highest, followed by Hst3 and then pHst1. Previous research has shown that Hst5 can adopt PPII-helix elements.^6^ The PPII-helix signal of Hst1 is deemed nonsignificant, although the interpretation is ambiguous.

**Figure 3:**
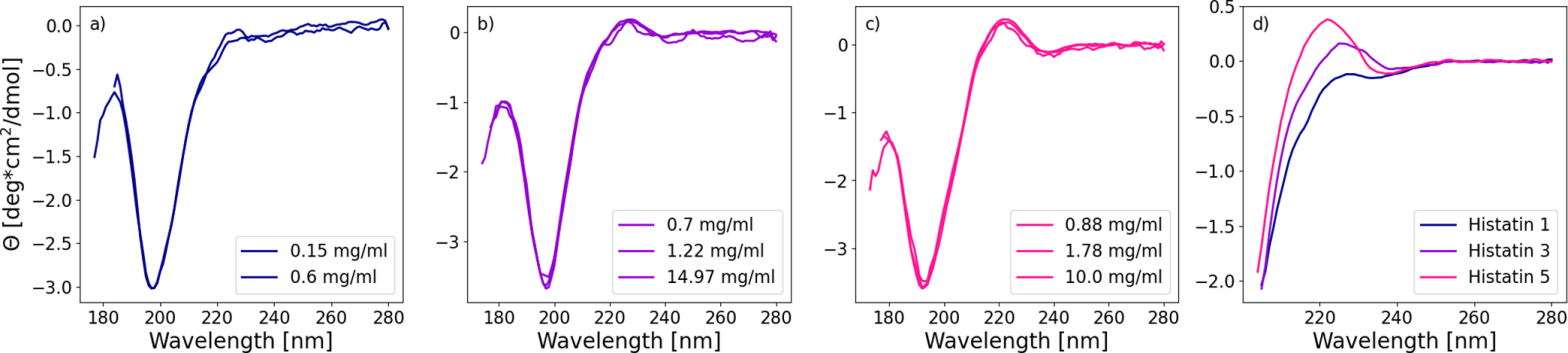
Circular dichroism spectra, molar ellipticity, Θ, as a function of wavelength, for Histatin 5 (c), Histatin 3 (b), and phosphorylated Histatin 1 (a). A comparison of the positive PPII-helix bands around 225 nm (d). Note the negative bands at 200 nm in parts (a)–(c), which signify disordered behavior.

The simulated conformations underwent decomposition based on *R*_ee_, *R*_g_, and *P*_S_; see Figure S1. As would be expected, *R*_ee_ and *R*_g_ increase with chain length. Notably, Hst1 exhibits a substantially narrower *R*_g_ distribution. This implies a tendency toward more compact structures, which matches the findings in Figure 2. DSSP^26^ was run on all of the decomposed groups; see Figure 4. Simulations approximately agree with the CD data, as most elements are considered bends, turns, or unordered. Unordered elements are defined as uncategorized in DSSP and are therefore not displayed in Figure 4. All simulations portray a significant occurrence of PPII-helix with an increased tendency at less compact conformations. As discussed in Figure 3, the experimental data generally do not support PPII-helix elements in pHst1, which indicates that the model over-represents such structural components in this case. It is unclear what caused the deficiency in the simulated data, DSSP, force field, simulation setup, etc. Nevertheless, there is a discrepancy between simulation and experiment. Further examination of the DSSP data indicates a heightened occurrence of other structural elements, such as 3–10-helix or β-bridge elements, in pHst1 and Hst3. Figure 4 also underscores how the choice of decomposition parameter affects data visualization. In the case of DSSP, it could be argued that a finer resolution of decomposition better displays trends within the data.

**Figure 4:**
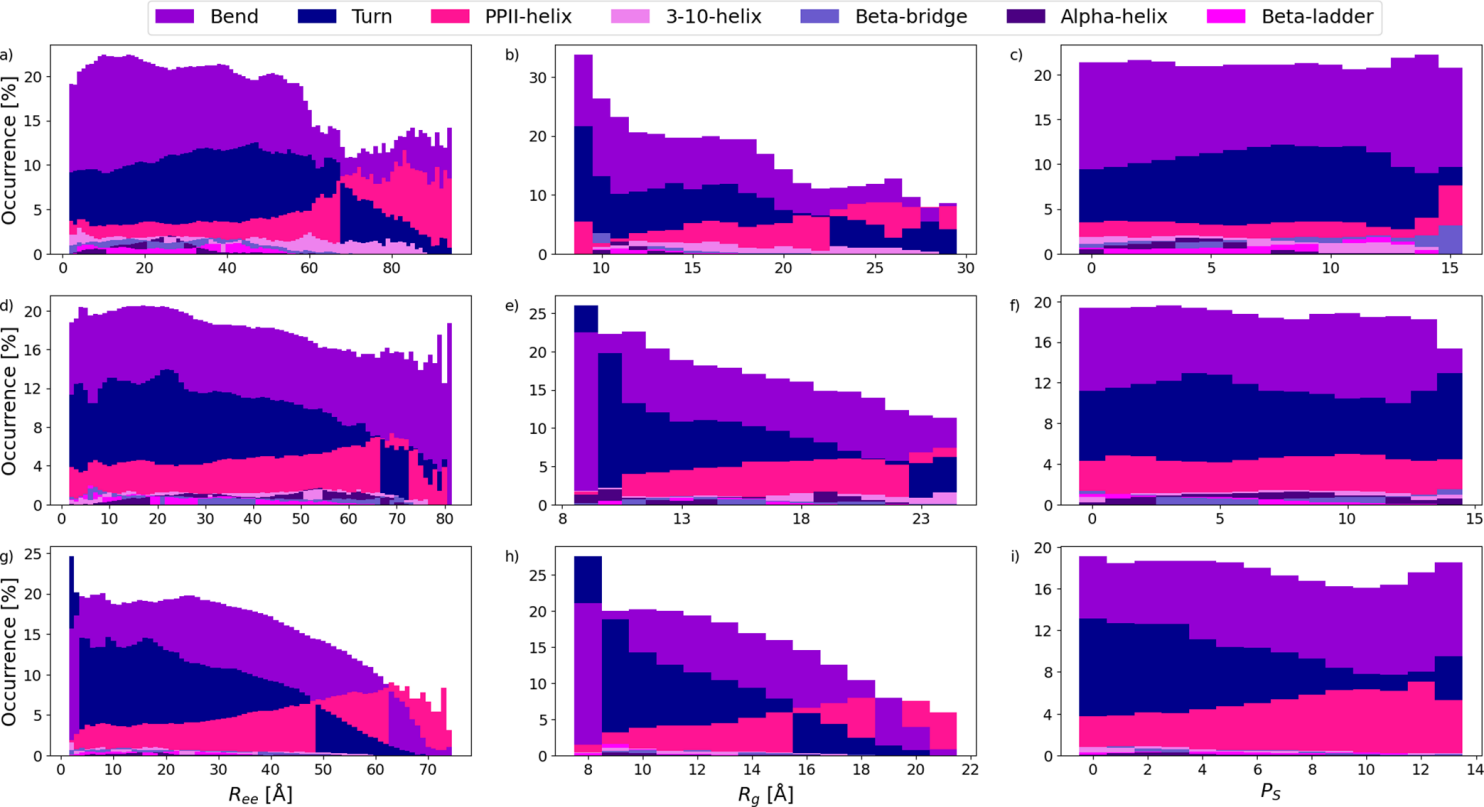
Decomposed DSSP data for the Histatin 5 (g, h, i), Histatin 3 (d, e, f), and phosphorylated Histatin 1 (a, b, c) simulations. Decomposition parameters included radius of gyration (*R*_g_) (a, d, g), end-to-end distance (*R*_ee_) (b, e, h), and polymer shape (*P*_S_) (c, f, i).

#### Free Energy Landscapes and Degree of Disorder

The variation within the conformational ensemble was contextualized using free energy landscapes. As would be expected, the three peptides exhibit excessively varied state distributions. Interestingly, Hst3 attains lower energy states than pHst1 and Hst5. Deeper free energy wells could signal a less disordered nature. AIUPred^27^ was used to gauge the degree of pHst1, Hst3, and Hst5 disorder. All mentioned analyses are shown in Figure 5. AIUPred assigns a probability of disorder to each residue within a submitted sequence. As seen in Figure 5d, the likelihood tends to decrease with chain length. The simulated average RMSF per residue matches this trend. Hst1 and Hst3 also decrease across the chain when approaching the C-terminus. Figure 2 shows no major difference in average shape other than a potentially higher tendency toward globular shapes in pHst1. However, SAXS data has a relatively low resolution. There exists evidence of a lower degree of structural flexibility in Hst3 when compared to Hst5, which was determined using nuclear magnetic resonance (NMR).^36^ Altogether, it appears likely that the three peptides could differ in degree of disorder, being mindful of the simulation shortcomings related to pHst1, which were discussed previously.

**Figure 5:**
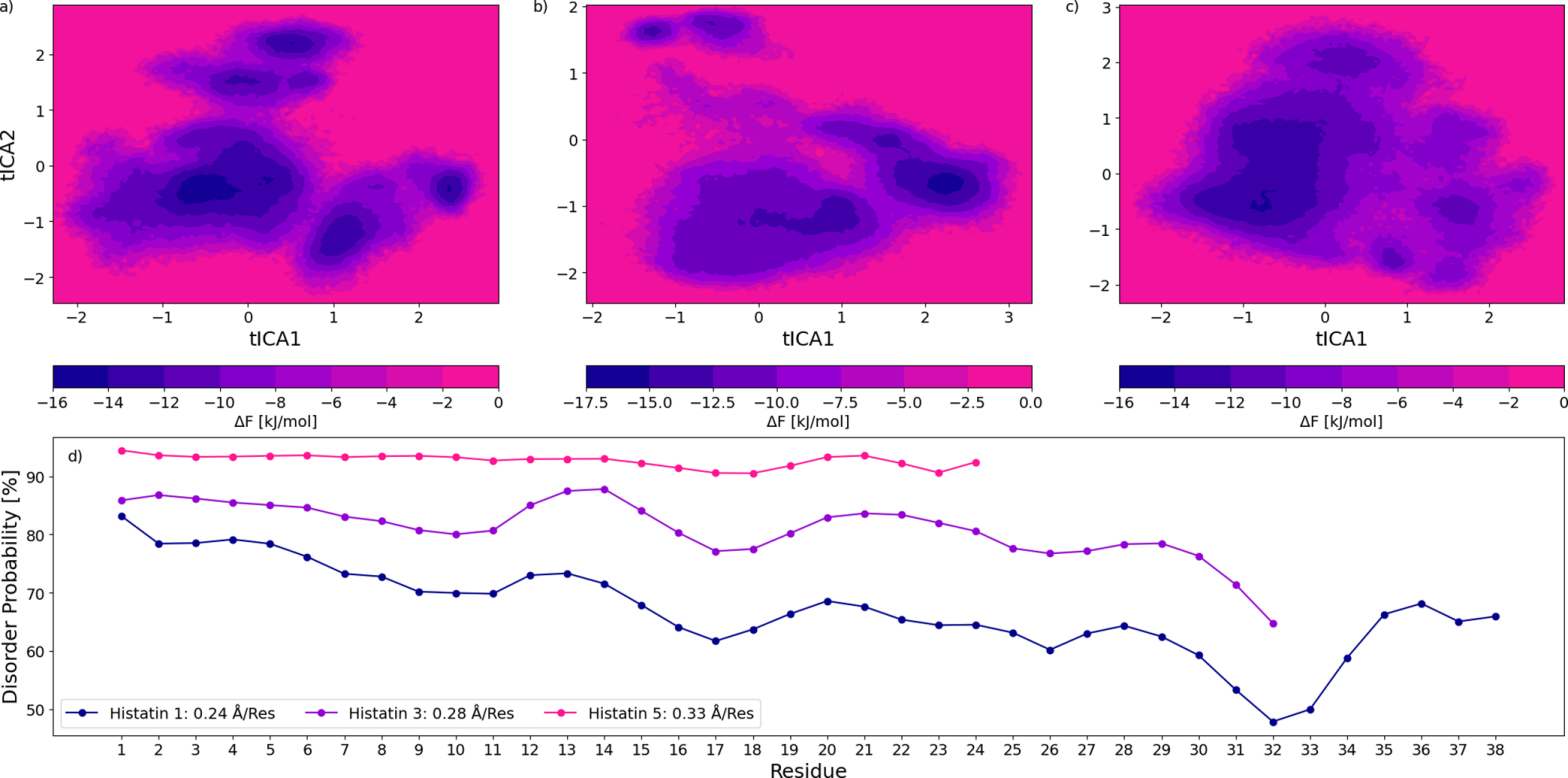
Free energy landscape for Histatin 5 (c), Histatin 3 (b), and phosphorylated Histatin 1 (a), as well as AIUPred determined disorder probabilities coupled with average root-mean-square fluctuations per residue (d). The free energy landscapes are based on a time-lagged independent component analysis (tICA) dimensionality reduction. Thus, the principal components act as axes.

### Phosphorylated Histatin 1

#### Analysis of the Conformational Ensemble through Multivariable Decomposition

Each simulated conformation *i* is characterized by its *R*_g_, *R*_ee_, and SASA values, designated as characteristic properties. By defining a summarized property deemed the conformation parameter, *X*_conf_, according to eq 4, a generalized decomposition can be performed. Where *R*_g,ave_, *R*_ee,ave_, and SASA_ave_ are the arithmetic average of the respective properties. The α parameter defines the resolution of the decomposition; in Figure 6, it was set to a value of five. The effects of α are explored further in Figure S6.

4 $$X_{\mathrm{conf},i}=\left[\frac{R_{g,i}}{R_{g,\mathrm{ave}}}+\frac{R_{\mathrm{ee},i}}{R_{\mathrm{ee},\mathrm{ave}}}+\frac{\mathrm{SAS}A_{i}}{\mathrm{SAS}A_{\mathrm{ave}}}\right]\alpha$$

**Figure 6:**
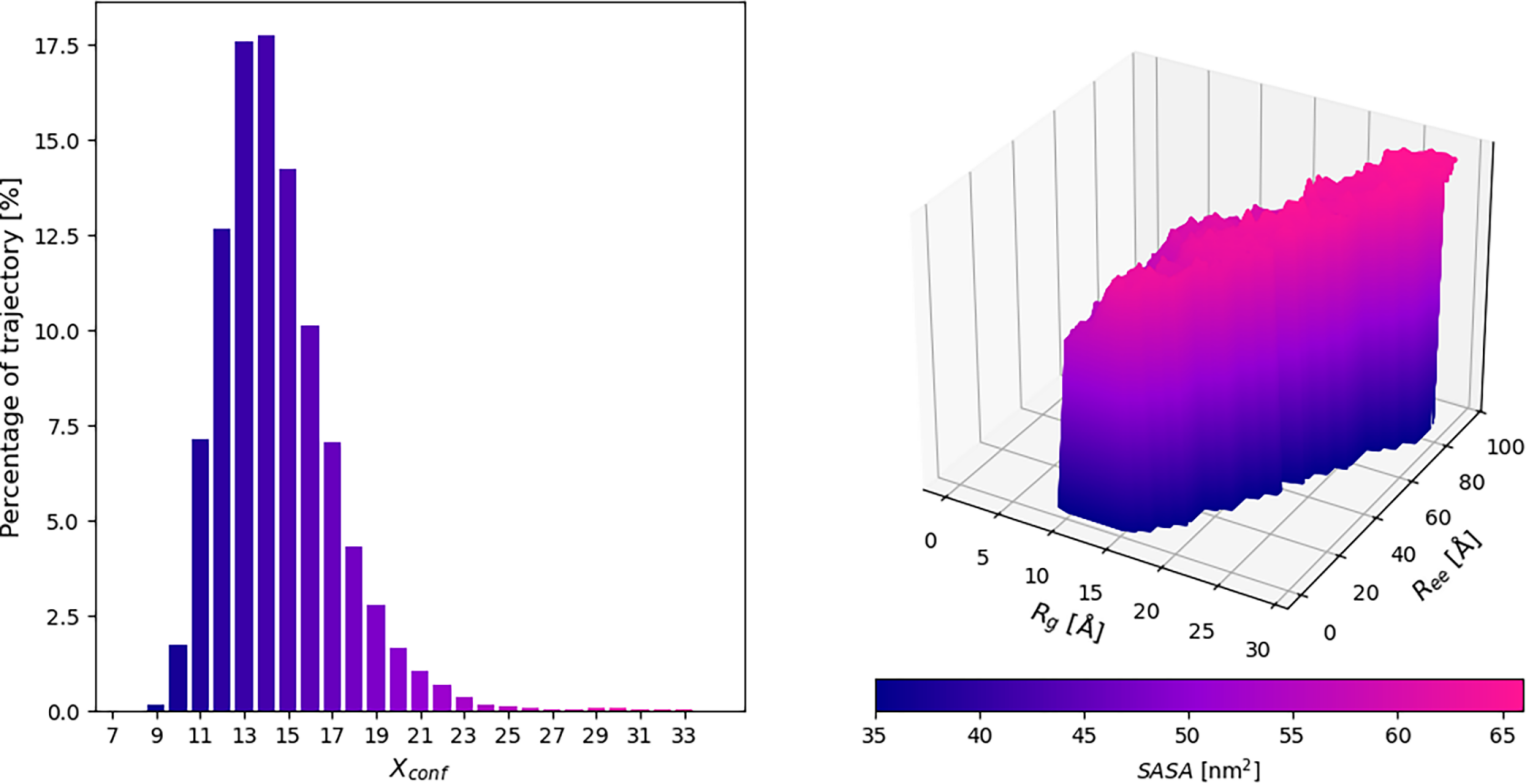
Linear proportionality of radius of gyration (*R*_g_), end-to-end distance (*R*_ee_), and solvent-accessible surface area (SASA) present within simulated conformations allow for decomposition based on the derived conformation parameter (*X*_conf_) applying the simulation averages.

Equation 4 is based on the proportional relation between the characteristic properties, which are presented in Figure 6 alongside the multivariable decomposition of the pHst1 simulation. As a result of the proportionality above, a higher *X*_conf_ typically indicates extended conformations with solvent accessibility, while a lower value indicates the opposite.

#### Intramolecular Interactions

The secondary structure data showcases the significant variety of intramolecular interactions. This was further examined using contact maps; see Figure 7. A contact map consists of a contour plot showing the number of hydrogen bonds per frame for every possible residue–residue pair within the chain. More hydrogen bonding occurs at lower *X*_conf_ values, which is visible in the number of hydrogen bonds per frame and the number of notable bonding pairs. Some bonding pairs persist in all three sampled groups. These are tabulated in Table 1 by calculating the average minimum pair distance within each respective *X*_conf_ group.

**Figure 7:**
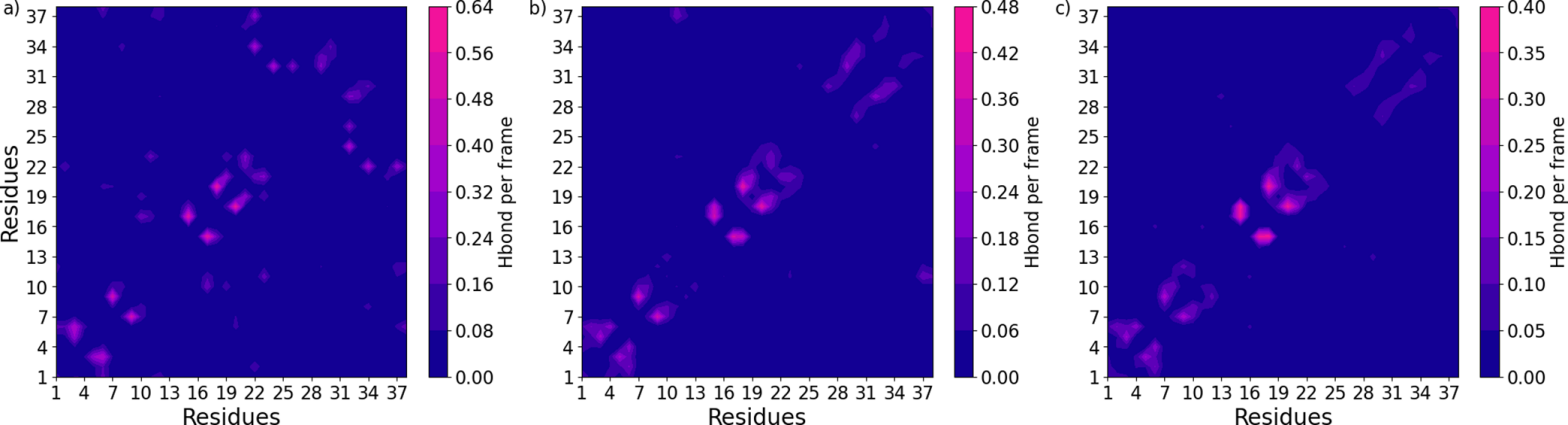
Hydrogen bond contact maps for three sampled conformational parameter (*X*_conf_) groups, 10 (a), 15 (b), and 20 (c). Each coordinate in a contact map represents a hydrogen bonding pair within the peptide chain. The color scheme’s intensity is coupled to the number of hydrogen bonds per frame. Notice how the intensity and number of noteworthy pairs increase by lowering *X*_conf_.

**Table 1:** Average Minimum Pair Distances for Consistently Occurring Hydrogen Bonding Pairs within the Sampled Conformational Parameter (*X*_conf_) Groups

	*X*_conf_ = 10	*X*_conf_ = 15	*X*_conf_ = 20
Lys5-His3	2.89 Å	3.05 Å	3.21 Å
Gly9-His7	2.57 Å	2.92 Å	3.25 Å
Lys17-His15	2.41 Å	2.83 Å	2.94 Å
His18-His15	4.04 Å	4.23 Å	3.95 Å
Ser20-His18	2.74 Å	2.91 Å	2.85 Å
Arg22-His21	1.33 Å	1.34 Å	1.34 Å
Ser32-Asp29	5.01 Å	5.59 Å	6.22 Å

Generally, the pairs display increasing distance with increasing *X*_conf_, although there are some exceptions. Arg22-His21 stays consistent in all sampled *X*_conf_ groups, likely due to the residues being neighbors. His18-His15 and Ser20-His18 showcase the largest distances at *X*_conf_ = 15.

#### Clustering and Example Structures

Clustering algorithms provide a natural complement to decomposition, allowing another option for sorting frames. Frames within each cluster can then be further analyzed following a similar protocol to decomposition. This was showcased via the extraction of an example structure. Frames of each of the further scrutinized *X*_conf_ groups were subjected to *K*-means clustering. Four clusters for each group were deemed optimal after applying the Elbow method. Figure 8 presents clusters and selected example snapshots. Each extracted structure is a representative average of the specific cluster. The snapshots show further breadth of conformational variation with examples of secondary structure element occurrence and variation of hydrogen bonding. Intricate hydrogen bonding networks involving the phosphoserine at position and key residue–residue pairs found in Table 1, such as Ser20-His18, are visible. A transient case of β-structure is also observed. Figure 8 underlines the natural connection between decomposition and clustering methods, such as *K*-means clustering.

**Figure 8:**
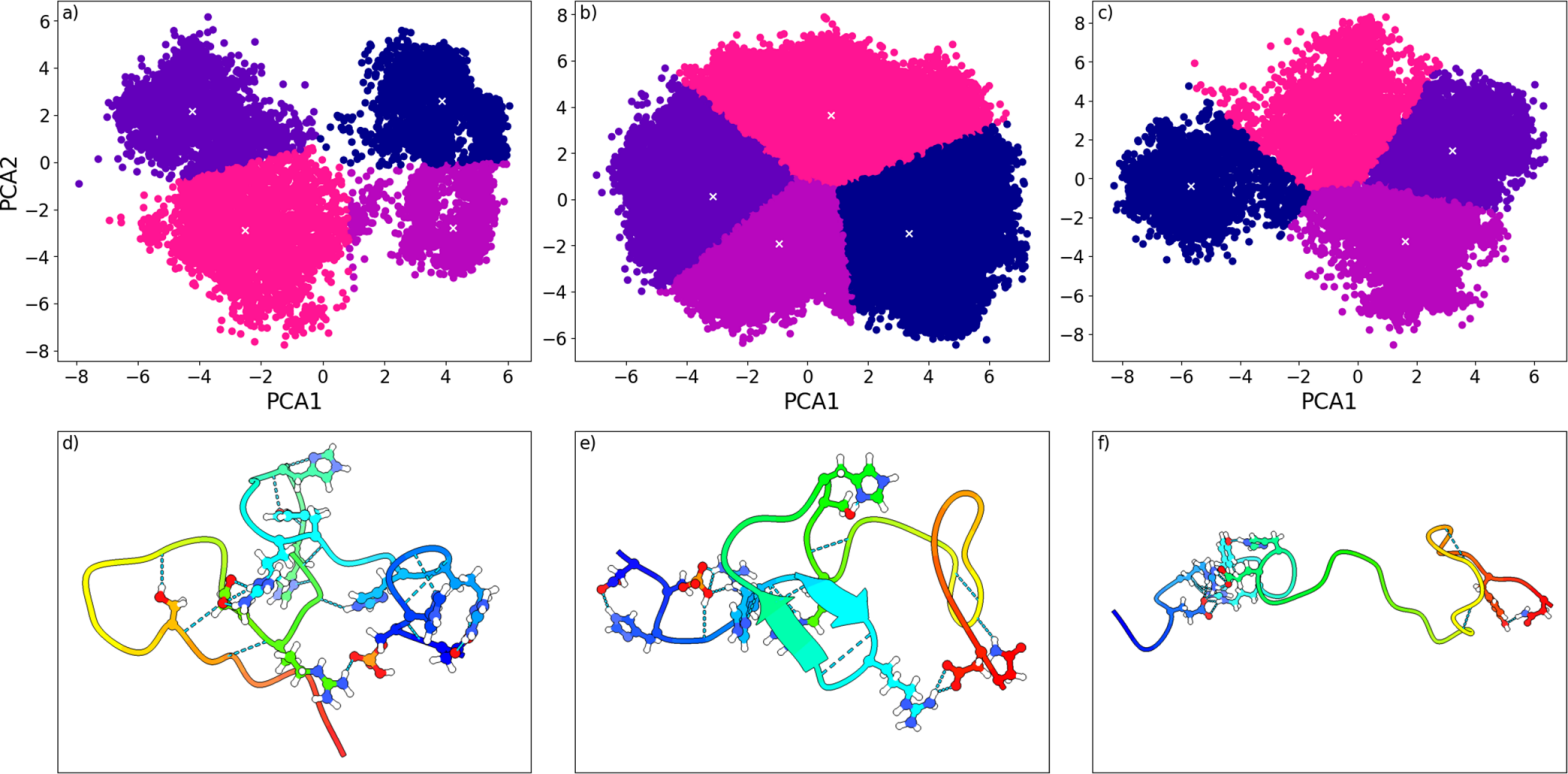
*K*-means cluster determined from the selected conformational parameter (*X*_conf_) groups,10 (a), 15 (b), and 20 (c), as well as example structures extracted from said clusters, 10 (d), 15 (e), and 20 (f). White crosses signify the centroid points. The data underwent a principal component analysis (PCA) dimensionality reduction. Thus, the principal components act as axes.

### Impact of Phosphorylation

#### Variation in Conformational Ensemble

An initial investigation on the difference in conformational ensemble was performed by determining the free energy landscape; see Figure 9a,b. The phosphorylation of the Ser2 position appears to significantly affect the conformational ensemble of Hst1, with a complete rearrangement of the free energy landscape. RMSF was calculated for each residue to examine how the phosphorylation affects movement within the chain, displayed in Figure 9c. A decrease in the fluctuation of the N-terminus section of the peptide is observed upon phosphorylation of Ser2, specifically the Asp1-Ser2-His3 metal binding motif. Figure 9d also displays a significant SASA increase for Ser2. This metal binding region is likely involved in the binding to tooth enamel.^2,5,38^ The connection between the phosphorylated Ser2 position and enamel binding has been indicated experimentally. For example, Yin et al. used a Langmuir-type adsorption model to show that adding phosphorylation increased affinity to hydroxyapatite.^39^ Based on the data in Figure 9, it seems possible that the phosphorylation shifts movement further down the chain and biases the conformational ensemble toward a decreased flexibility and solvent-accessible N-terminus to facilitate binding, potentially through electrostatic interaction. The decreased fluctuations of Asp1-Ser2-His3 appear to connect with an increase in some centrally located residues, residues 10 through 18. Residues 10 through 18 contain parts of another metal binding motif, His15-Glu16-Lys17-His18-His19.^38^ Such shifting can be further showcased using minimum distance contact maps; see Figure 10. It is observed that there is an apparent change between the residue–residue pairs surrounding the middle region of Hst1. The nonphosphorylated state exhibits several points of more significant distance within the contact map, which have disappeared in the phosphorylated map. This observation connects to the changes in fluctuations seen in Figure 9 since the points of more significant distance overlap decently well with the His15-Glu16-Lys17-His18-His19 region.

**Figure 9:**
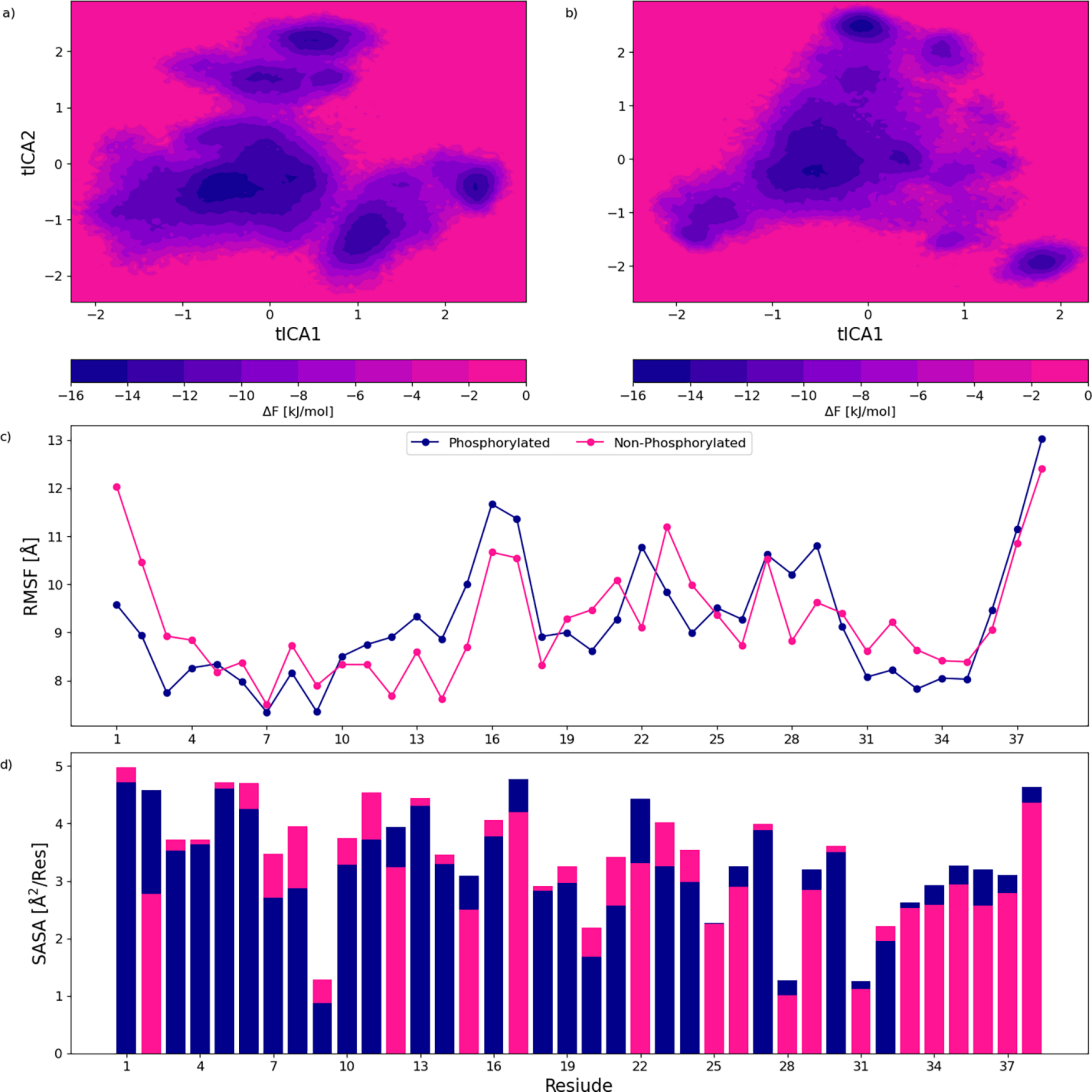
Free energy landscapes for the phosphorylated (a) and nonphosphorylated states of Histatin 1 (b). Root-mean-square fluctuations (RMSF) per residue (c) and normalized solvent accessible solvent area (SASA) per residue (d) for both states. The free energy landscapes are based on a time-lagged independent component analysis (tICA) dimensionality reduction. Thus, the principal components act as axes.

**Figure 10:**
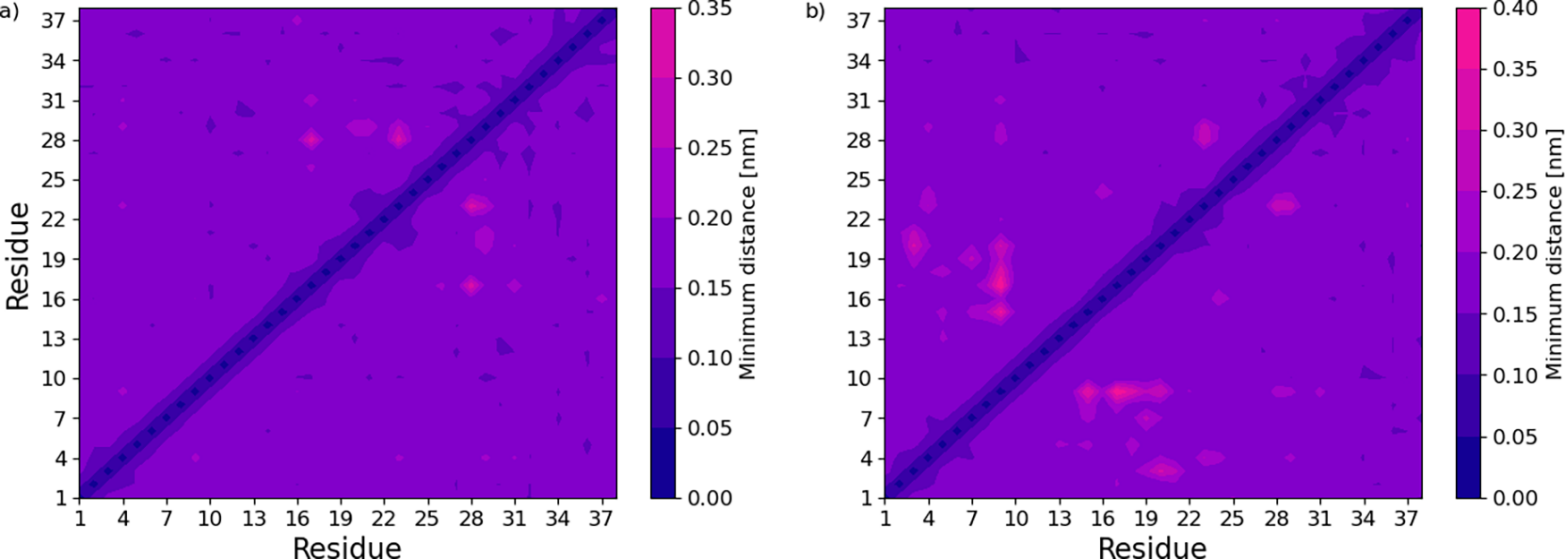
Minimum pair distance contact maps of phosphorylated (a) and nonphosphorylated Histatin 1 (b). Each point on the maps represents a residue–residue pair within the peptide chain. The color scheme showcases the minimum distance observed throughout the simulation, normalized for both plots to range between zero and the highest recorded distance, 0.40 nm. Note how noteworthy points fluctuate between plot (a) and (b).

#### Phosphorylation-Induced Secondary Structure

Results of Figure 9 indicate that the phosphorylation could act as a molecular switch that biases the conformational ensemble toward more structures that more readily bind to enamel. The potential of phosphorylation-induced secondary structure was analyzed through kernel density estimations involving DSSP^26^ data and *R*_ee_; see Figure 11. The propensity for 3–10-helix and β-bridge elements remains identical, mainly between the two states.

**Figure 11:**
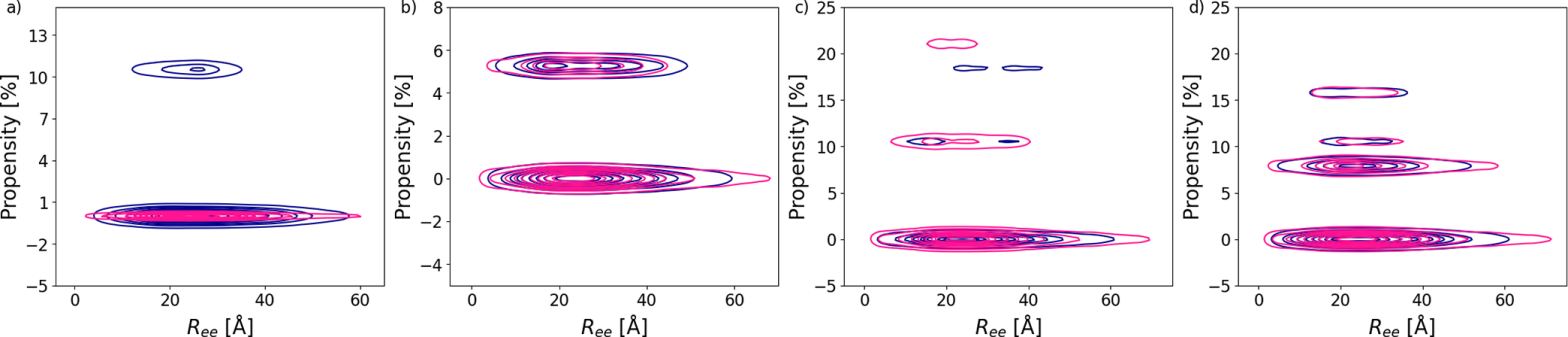
Secondary structure propensity versus end-to-end distance (*R*_ee_) displayed as kernel density estimations, α-helix (a), β-bridge (b), β-ladder (c), and 3–10-helix (d). Data about phosphorylated Histatin 1 is dark blue, and nonphosphorylated Histatin 1 is deep pink.

α-Helix and β-ladder elements show apparent variation. These element types were further scrutinized on a residue level in Figure 12. Hst1 has two pronounced helical sections, residues 2 through 8 and 28 through 37. β-ladder is centered on the C-terminus, particularly Tyr30, Gly31, Tyr34, and Leu35. Upon phosphorylation, most α-helical elements occur in the residue 28 through 37 region, and β-ladder elements spread out more across the chain, which we hypothesize provides evidence that phosphorylation is a molecular switch, though purely computationally.

**Figure 12:**
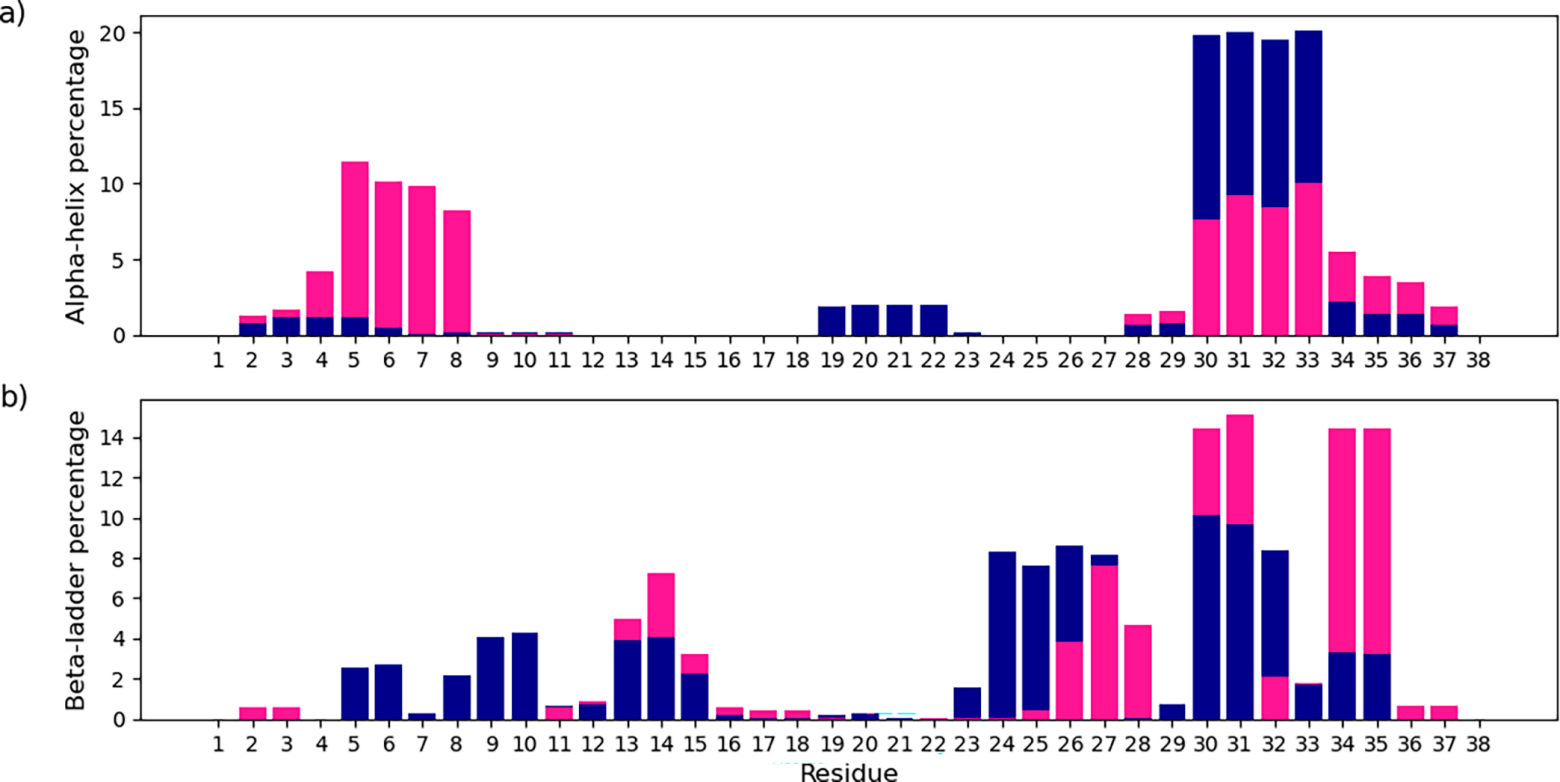
α-Helix (a) and β-ladder (b) element occurrence on a residue level. The total number of occurrences of the specific structural element has normalized the occurrence of each residue. Phosphorylated Histatin 1 is dark blue and nonphosphorylated Histatin 1 is deep pink.

### Antimicrobial Killing Mechanisms of Histatins

The current consensus on the antifungal killing mechanism of Hst5 includes the passage of the peptide through the cell membrane and targeting of the mitochondria.^7,40^ There is also a precedent for membrane interactions being mainly driven by electrostatics, more specifically, a need for an internal negative potential for the peptide to be active against a target.^40^ The intensely disordered nature of Hst5, which has been showcased herein and in previous research,^6,7,12^ could signal a high preference toward polar electrostatically favorable environments. As depicted in Figure 5 and additionally supported by NMR measurements by Brewer et al., this may be diluted in Hst3 and pHst1, perhaps due to the addition of hydrophobic residues. This is further contextualized when considering Hst1 in full since the chain displays two distinct electrostatically differing regions: residues 1 through 22, which are highly polar, and residues 23 through 38, which are hydrophobic. From these structural observations, it could be hypothesized that Hst1 differs from Hst3 and Hst5 due to self-association driven by hydrophobic interactions between C-terminus regions. Signs of association were measured with SAXS; see Figure 13. The apparent increase in intensity seen at a low *q-*value within Figure 13a indicates a self-associated system. Furthermore, the *R*_g_ increases from 15.8 to 27.2 Å.

**Figure 13:**
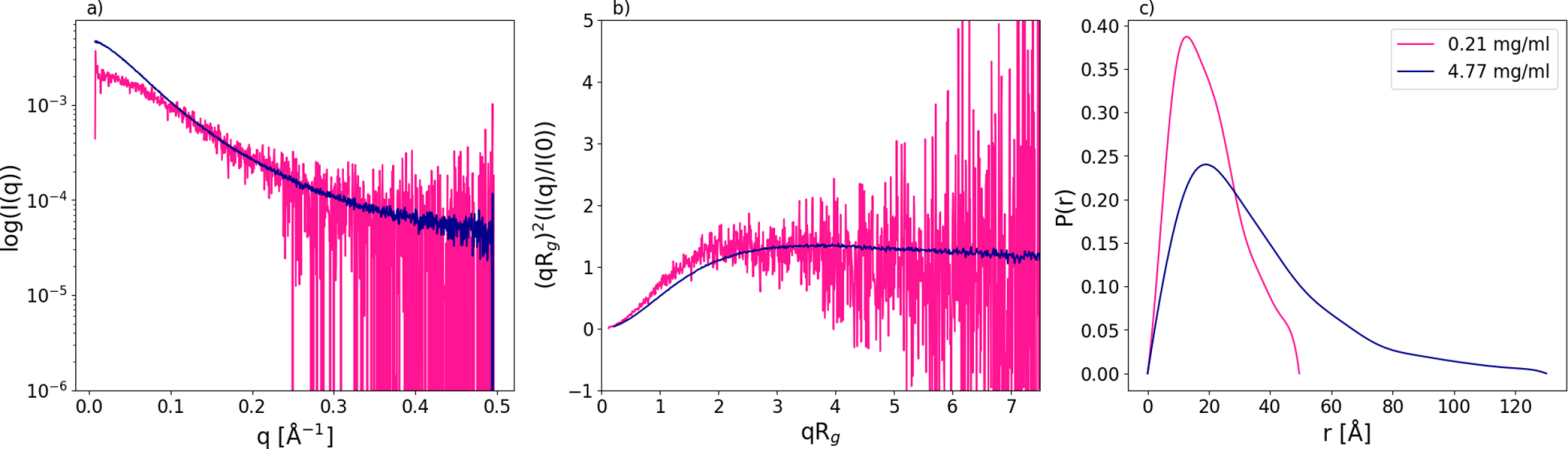
Small-angle X-ray scattering spectra of phosphorylated Histatin 1 at a concentration of 0.21 and 4.77 mg mL^–1^. The intensity, *I*(*q*), as a function of the scattering vector, *q*, on a semilogarithmic scale (a) and normalized Kratky plots, (*qR*_g_)^2^(*I*(*q*)/*I*(0)), as a function of *qR*_g_ (b), and a pair distance distribution function, *P*(*r*) (c). Note that the spectra have been normalized in *q* = 0.1.

The existence of self-association and Hst1’s potentially amphipathic character has profound effects when contrasting the antifungal and antiviral properties of Hsts. The membrane interaction of Hst3 and pHst1 was investigated through NR. Hst3 (data not shown) was determined to behave similarly to Hst5 and some of its variants already investigated by us and reported in the literature.^6,41^ On the contrary, pHst1 dramatically affects the SLB, which has not been observed for any variant investigated. The analysis of NR data, resulting in the SLD profiles reported in Figure 14a, supports a 2-fold process. Hst1 molecules accumulate near the solid substrate, forming a hydrated peptide layer 4.5 nm thick. In the SLD profiles, a signature of the presence of an SLB is still visible but shifted toward larger distances from the substrate (*z* ≈ 60–100 Å). However, the significant split between the profiles is due to water molecules occupying an overall 35% of the SLB hydrophobic region, which implies that 35% of the lipid molecules were removed for the unperturbed SLB. Figure 14a shows the SLD profile with a dashed black line. Above the remaining portion of the SLB, it was possible to detect the presence of a diffuse and nonstructured layer composed of, likely, peptides and lipids. Notably, due to the significant effect on the SLB structure and the mixing of different molecular components, a more quantitative data analysis could not be performed, as no model typically used for SLBs was applied. However, the results are unambiguous and suggest that Hst1 might attack microbes via membrane rupturing, perhaps similar to other antimicrobial peptides such as Melittin.^42,43^ Melittin’s precise mechanism is elusive. However, central concepts of its mode of action are its amphipathic nature and accumulation of peptide.^42,43^ Figure 14c,d shows that Hst1 mimics Melittin since its trans-membrane tendency and hydrophobicity are skewed to specific sections of the chain, albeit to a lesser extent. Antimicrobial action through membrane disruption is a commonly seen phenomenon. In addition to Melittin, Magainin 2,^44^ LL-37,^45^ and Cecropin A^46^ are some more examples of peptides that operate via lipid bilayers. A CIDER analysis^29^ was carried out on Hst1, Melittin, Magainin 2, LL-37, and Cecropin A, see Table 2. Table 2 is noteworthy as it exemplifies various characteristics within membrane-active peptides. However, specific trends seem consistent, such as high fraction of charged residues (FCR), high hydropathy, and high fraction of disorder-promoting residues. The culmination of the gathered data proposes that the antiviral killing mechanism of Hsts could be wholly unlike the antifungal mechanism. The carpet model is a potential candidate for the mechanism.

**Figure 14:**
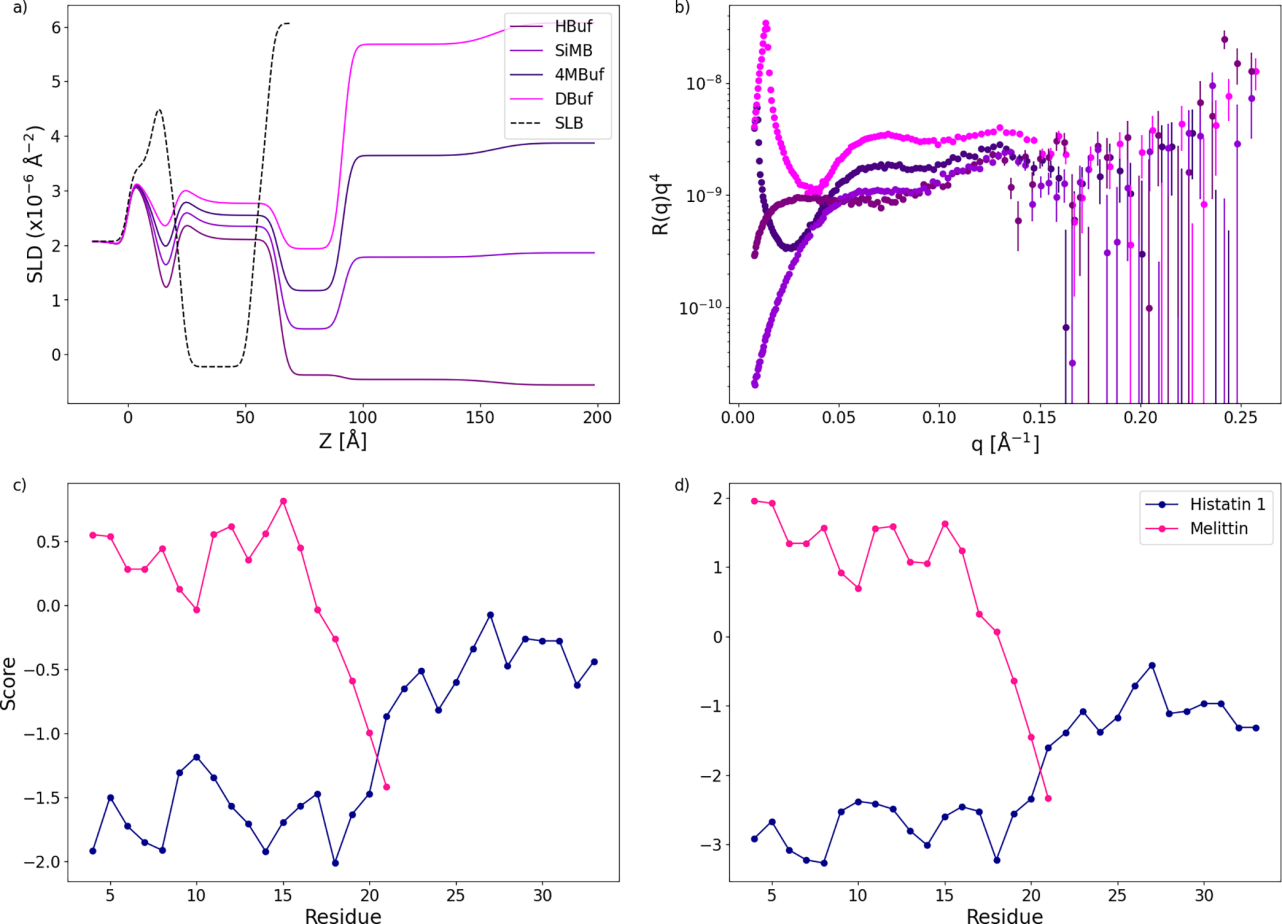
Scattering length density (SLD) curves (a) and reflectivity plots (b), determined from neutron reflectivity measurements of phosphorylated Histatin 1. HBuf, DBuf, 4MBuf, SiMB, and SLB signify 100% H_2_O, 100% D_2_O, 66/34 ratio D_2_O/H_2_O, 38/62 ratio D_2_O/H_2_O, and the unperturbed supported lipid bilayer (SLB), respectively. Calculated trans-membrane tendency (c) and hydrophobicity (d) per residue for Melittin and nonphosphorylated Histatin 1.

**Table 2:** CIDER Analysis for Histatin 1, LL-37, Cecropin A, Melittin, and Magainin 2^a^

peptide	length	κ	FCR	NCPR	hydropathy	disorder promoting
Hst1	38	0.179	0.342	0.026	2.405	0.711
LL-37	37	0.092	0.432	0.162	3.776	0.622
Cecropin A	37	0.142	0.270	0.162	4.427	0.649
Melittin	26	0.616	0.192	0.192	4.773	0.615
Magainin 2	23	0.450	0.217	0.130	4.583	0.609

a Columns consist of peptide length, the extent of charge mixing (κ), the fraction of charged residues (FCR), the net charge per residue (NCPR), hydropathy, and the fraction of disorder-promoting residues.

In summary, Hst3 and Hst5 are likely to function through seamless membrane translocation and mitochondrial targeting.^7,40^ In contrast, Hst1 may employ a distinct mechanism involving membrane disruption, which could account for its lower antifungal potency. Such a difference also provides valuable insight into the diverse modes of action that may underlie the antiviral properties of Histatins. Further investigation into Hst1’s potential for self-association and membrane interaction is warranted and currently underway.

## Conclusions

This study provides a detailed view of the structural comparison between Hst1, Hst3, and Hst5, as well as conformational variations caused by the phosphorylation of Hst1. Structural differences between the peptides were classified through a highly integrated approach, applying multiple experimental and computational techniques. These variations could be mechanistically crucial for their antimicrobial properties since evidence was provided regarding the concepts of self-association and the membrane rupturing mechanism of Hst1. A novel multidimensional decomposition technique was presented and explored. The crucial link between decomposition and clustering was also exemplified. It was shown how the phosphorylation of the Ser2 position directly shifts the conformational ensemble toward conformations with a solvent-accessible and less flexible N-terminus region. The data indicate that the phosphogroup could act as a molecular switch that skews the conformational ensemble to facilitate binding to tooth enamel. This is supported by clear changes in secondary structure elements upon modification. While the presented findings are valuable, simulated data of pHst1 was produced with a model that does showcase certain shortcomings, which should be openly offered.

## Scientific Novelty

1. The decomposition method was extended into a multidimensional form with the possibility to set the resolution. The potential ties to clustering algorithms were also explored.
2. Structural differences between Hsts were showcased and investigated, most prominently, Hst1’s potential ability to self-associate and the possibility that it acts through membrane rupturing.
3. This paper provides an extensive example of structural biology research using an integrative approach, applying both experiments and computational methods.

## Supplementary Material



## Data Availability

Neutron reflectometry data are stored at https://doi.ill.fr/10.5291/ILL-DATA.8-02-1007. All MD simulation trajectories are available upon request. All input files to run the simulations, the script implemented for the decomposition, as well as figure scripts, can be downloaded from the GitHub repository: https://github.com/oskasve/Histatin-1-and-its-Siblings.

## Notes

Convergence analysis for the simulations, further explorations of the α parameter, and decomposition distributions of *R*_g_, *R*_ee_, and *P*_S_ (PDF)
