# Supplementary material for: Multidimensional Decomposition and Ensemble Modeling of Histatin 1 and Its Siblings: Detailing Structure and Biological Function Using an Integrative Approach
Source: J Chem Inf Model. 2025 Jul 2;65(13):7089–101. doi: 10.1021/acs.jcim.5c00854 (PMC12264947; doi:10.1021/acs.jcim.5c00854)
Supplement: Supplementary file 1 [file ci5c00854_si_001.pdf]

Supplementary Information for Multidimensional Decomposition and Ensemble Modeling of Histatin 1 and Its Siblings: Detailing Structure and Biological Function using an Integrative Approach

Oskar Svensson,[†,‡] Yuri Gerelli,[¶,§] and Marie Skepö[*,†,‡]

†*Division of Computational Chemistry, Department of Chemistry, Science for Life Laboratory, Lund University, P.O. Box 124, SE-221 00, Lund, Sweden*

‡*NanoLund, Lund University, Box 118, 22100 Lund, Sweden*

¶*CNR - Institute for Complex Systems, Piazzale Aldo Moro 2, 00185 Roma, Italy*

§*Department of Physics, Sapienza University of Rome, Piazzale Aldo Moro 2, 00185 Roma, Italy*

E-mail: marie.skepo@compchem.lu.se

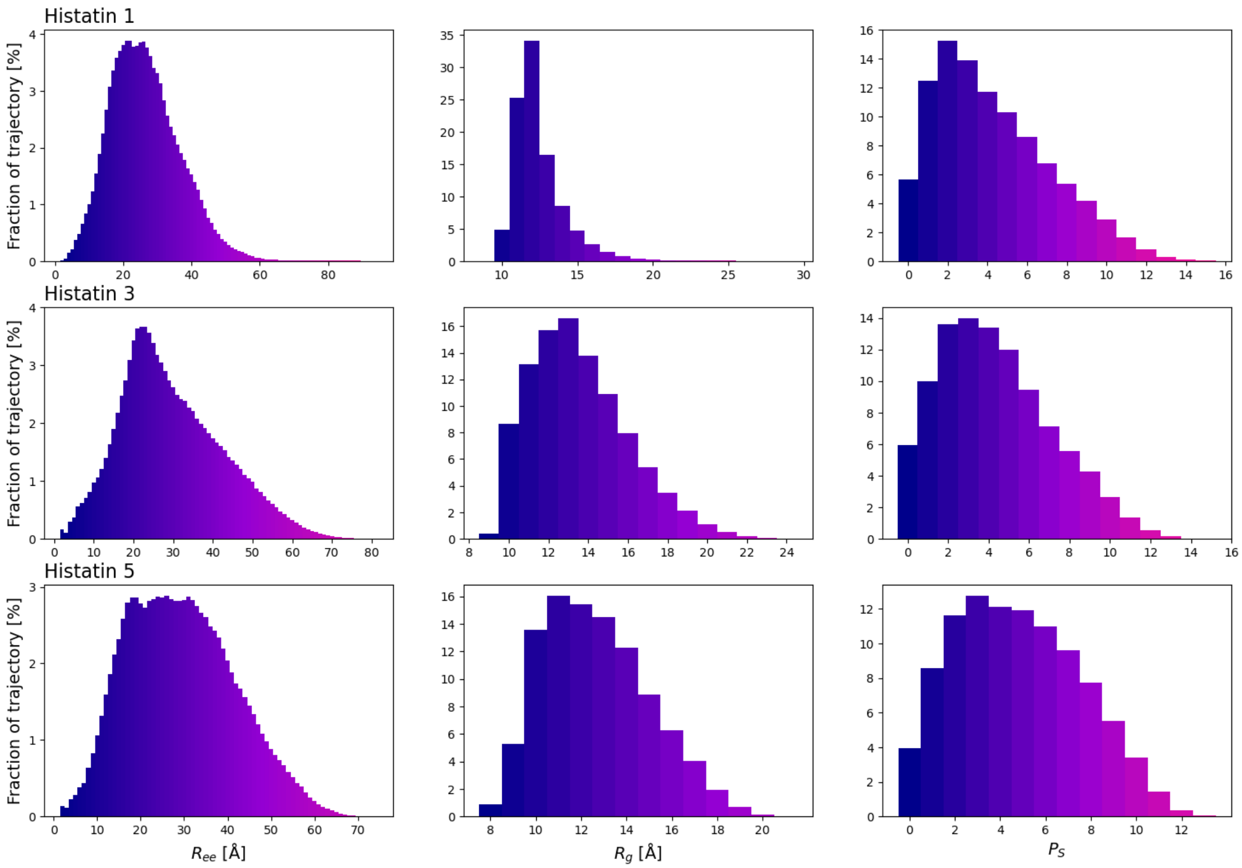

Figure S1: Decompositions of Histatin 5, Histatin 3, phosphorylated Histatin 1 simulated conformations based on end-to-end distance (R_{ee}), radius of gyration (R_g), and polymer shape (P_S).

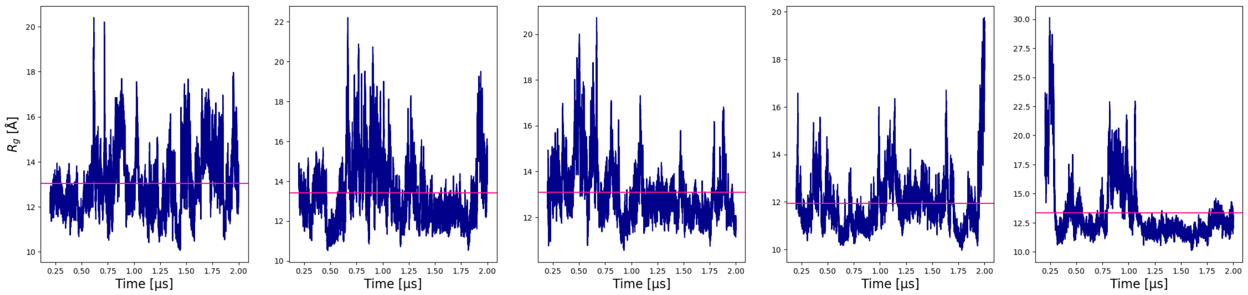

Figure S2: Convergence analysis for the phosphorylated Histatin 1 simulations.

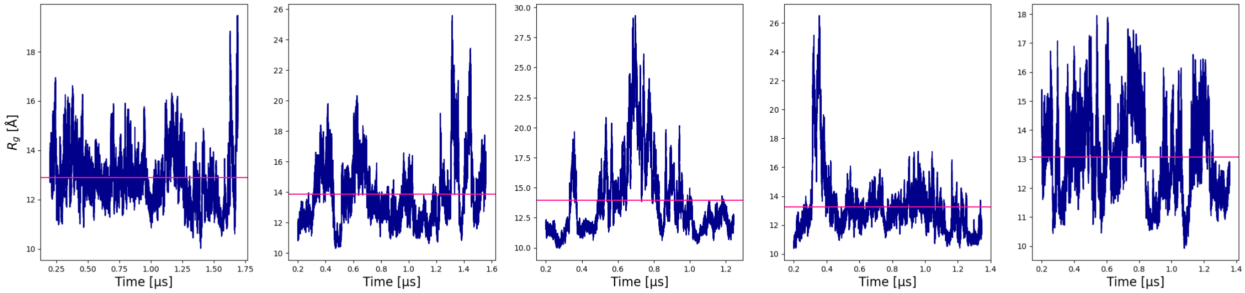

Figure S3: Convergence analysis for the non-phosphorylated Histatin 1 simulations.

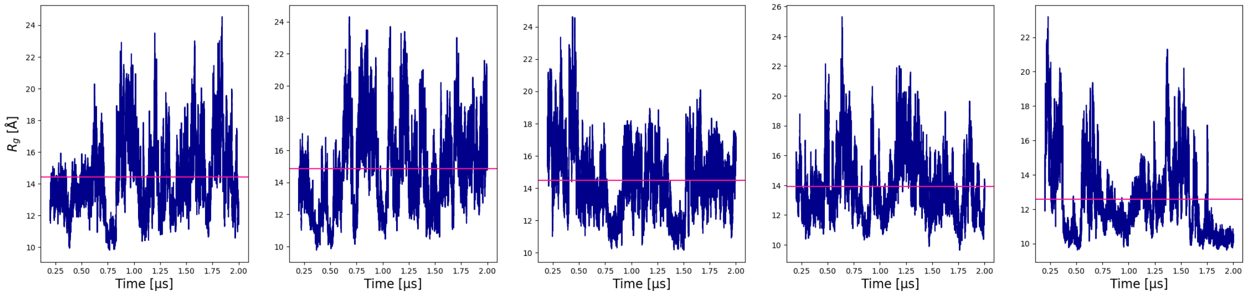

Figure S4: Convergence analysis for the Histatin 3 simulations.

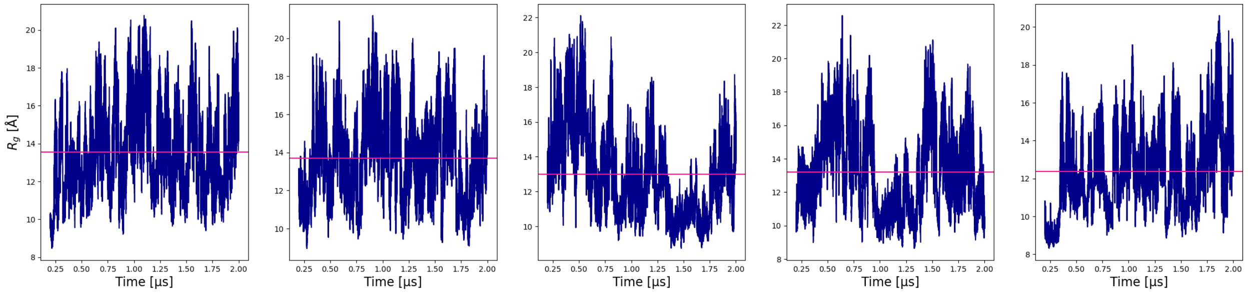

Figure S5: Convergence analysis for the Histatin 5 simulations.

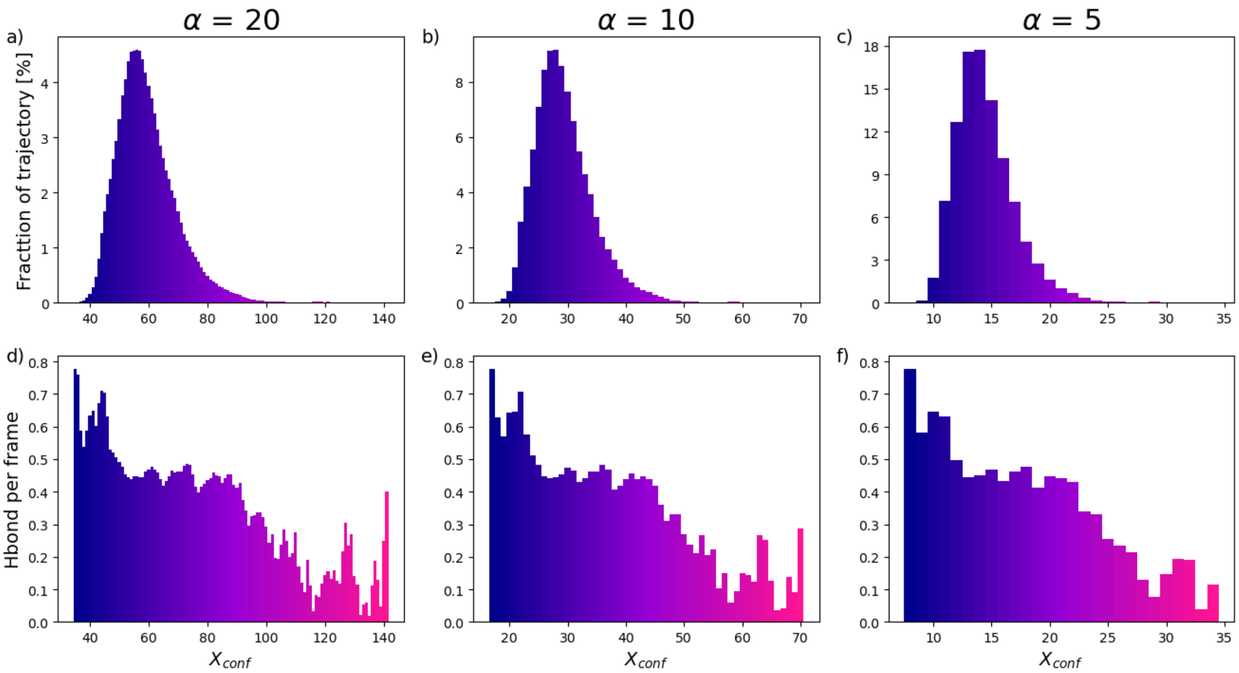

Figure S6: Multi-variable decompositions with three different α values (a-c). Number of hydrogen bonds per frame between SER2 and the rest of the peptide as a function of the confrontational parameter, X_{conf} (d-f).
